# Ready for a world without antibiotics? The Pensières Antibiotic Resistance Call to Action

**DOI:** 10.1186/2047-2994-1-11

**Published:** 2012-02-14

**Authors:** Jean Carlet, Vincent Jarlier, Stephan Harbarth, Andreas Voss, Herman Goossens, Didier Pittet

**Affiliations:** 1 Consultant, WHO African Partnerships for Patient Safety, 9 rue de la Terrasse, 94000 Créteil, France; 2 UPMC University Paris 6 EA, 154 Laboratory of Bacteriology-Hygiene and Microbiology, Hôpital Pitié-Salpétrière, Assistance Publique des Hôpitaux de Paris, 47-83 Boulevard de l'Hôpital, 75013 Paris, France; 3 Infection Control Programme and WHO Collaborating Centre on Patient Safety, University of Geneva Hospitals and Faculty of Medicine, 4 Rue Gabrielle-Perret-Gentil, 1211 Geneva 14, Switzerland; 4 Canisius-Wilhelmina Ziekenhuis and Radboud University Medical Centre, NTPDRD189, Postbus 9015, 6500 GS, Nijmegen, The Netherlands; 5 Laboratory of Medical Microbiology, University Hospital Antwerp, Wilrijkstraat 10, 2650 Edegem, Belgium; 6 3rd World Forum on Healthcare-Associated Infections, Annecy, France, 27-29 June 2011

**Keywords:** antibiotic resistance, antibiotic stewardship, infection control, hand hygiene, surveillance networks, care bundles, environment, regulations, human medicine, animal medicine

## Abstract

Resistance to antibiotics has increased dramatically over the past few years and has now reached a level that places future patients in real danger. Microorganisms such as *Escherichia coli *and *Klebsiella pneumoniae*, which are commensals and pathogens for humans and animals, have become increasingly resistant to third-generation cephalosporins. Moreover, in certain countries, they are also resistant to carbapenems and therefore susceptible only to tigecycline and colistin. Resistance is primarily attributed to the production of beta-lactamase genes located on mobile genetic elements, which facilitate their transfer between different species. In some rare cases, Gram-negative rods are resistant to virtually all known antibiotics. The causes are numerous, but the role of the overuse of antibiotics in both humans and animals is essential, as well as the transmission of these bacteria in both the hospital and the community, notably via the food chain, contaminated hands, and between animals and humans. In addition, there are very few new antibiotics in the pipeline, particularly for Gram-negative bacilli. The situation is slightly better for Gram-positive cocci as some potent and novel antibiotics have been made available in recent years. A strong and coordinated international programme is urgently needed. To meet this challenge, 70 internationally recognized experts met for a two-day meeting in June 2011 in Annecy (France) and endorsed a global call to action ("The Pensières Antibiotic Resistance Call to Action"). Bundles of measures that must be implemented simultaneously and worldwide are presented in this document. In particular, antibiotics, which represent a treasure for humanity, must be protected and considered as a special class of drugs.

## Background

In the golden age of the discovery of antibiotics, these potent "miracle" drugs saved millions of lives. In contrast, we are entering an era where bacterial infections, such as bloodstream infections and ventilator-associated pneumonia, might no longer be successfully treated with antibiotics [1]. We now face a dramatic challenge resulting from two combined problems. First, microorganisms are becoming extremely resistant to existing antibiotics, in particular Gram-negative rods (e.g., *Escherichia coli, Salmonella *spp, *Klebsiella *spp, *Pseudomonas aeruginosa, Acinetobacter *spp), which are resistant to almost all currently available antibiotics in some settings. Resistance can be combined with virulence, acting as a potentially deadly duo, as observed in the recent large epidemic outbreak of *E. coli *0104:H4 in Europe, notably in Germany [2]. Second, the antibiotic pipeline has become extremely dry [3]. Several new powerful compounds active against Gram-positive cocci have been made available in the last few years, but this is not the case for Gram-negative bacteria and almost no new antibiotic class active against multiresistant Gram-negative rods can be anticipated in the near future. Although hard to imagine, the reality is that many clinicians will soon face a therapeutic dead end in the treatment of certain types of severe bacterial infections. This worrisome situation takes us back to the pre-antibiotic era of the 1930s and early 1940s [1,3,4]. We cannot look at this evolving and pandemic threat passively and lose one of the most important drugs discovered in the previous century. We must act now; silence is not an answer.

In this position paper, we summarize important messages and conclusions from the 3^rd ^World Healthcare-Associated Infections (HAI) Forum held in June 2011. The meeting gathered together 70 leading world experts and opinion leaders in the domain of antimicrobial resistance (AMR) from 33 countries to discuss the challenges and possible options to tackle the problem. The main objectives were to structure and propose a hierarchy of the various measures reported in the recent literature and to collect information on the experiences of the many countries represented to discuss if some may be transposable to other nations.

### What are the facts about AMR?

Many alarming facts regarding AMR have accumulated, particularly over the last few years.

-- An increase in global resistance rates in many bacterial species responsible for both community- and healthcare-related infections, e.g., staphylococci, enterococci, gonococci, and enterobacteria (including *E. coli, Salmonella *spp and *Shigella *spp), *Pseudomonas *spp, *Acinetobacter *spp, and *Mycobacterium tuberculosis*) [1,5-7].

-- The burden of bacteremias due to *E. coli*, one of the most common human pathogens, is increasing in Europe, mainly due (but not only) to resistant strains [7].

-- Emergence and dissemination of new mechanisms of resistance, e.g., novel extended-spectrum beta-lactamases (ESBL) and carbapenemases [8-12]. The spread of the new resistance gene, the New Delhi metallo-beta-lactamase 1 (NDM-1), or other carbapenemases in *Enterobacteriacae *is alarming because these "superbugs" are resistant to most available antibiotics and can disseminate worldwide very rapidly, in particular as a consequence of medical tourism [12].

-- The rapid increase in the multiresistance of Gram-negative rods stands in contrast to a steady decrease in methicillin-resistant *Staphylococcus aureus *(MRSA) rates following the implementation of successful infection control programmes in several high-income countries, such as Belgium, France, United Kingdom (UK), and the USA [13-15]. In some other countries, resistance to both Gram-positive and -negative bacteria is very high (USA for community-acquired (CA)-MRSA; Greece, Italy, Portugal, UK, the USA, and many eastern European and Asian countries for vancomycin-resistant enterococci [VRE]).

-- Propensity to use last-line therapy (e.g., carbapenems) to treat healthcare-related and community-acquired infections triggered by a fear of infections caused by ESBL-producing *Enterobacteriaceae*, despite the fact that these antibiotics should be preserved as our last weapons against multiresistant Gram-negative bacteria.

-- Re-use of old drugs with poor safety and efficacy profiles and uncertain pharmacokinetic/pharmacodynamic characteristics (e.g., colistin) due to a lack of alternative drugs [16].

-- High morbidity and mortality attributable to multiresistant bacteria in critically ill patients.

• In Europe, the European Centre for Disease Prevention and Control (ECDC) reported that 25,000 people die each year from antibiotic-resistant bacteria [17].

• In the USA, MRSA is associated with a staggering 90,000 infections and an estimated 19,000 deaths annually [18].

-- Serious financial consequences of bacterial resistance.

• Multidrug-resistant organisms (MDROs) result in massive extra healthcare costs and productivity losses of at least 1.5 billion euros each year in Europe [17].

• In the USA, the annual cost of AMR in hospitals is estimated at more than US$ 20 billion with an even wider clinical impact than human immunodeficiency virus (HIV)-related disease [19].

However, these data on morbidity, mortality, and cost must be considered with caution and may be over- or underestimated because of a lack of in-depth adjustment for risk factors or evaluation of the indirect costs of AMR. Moreover, these figures were calculated before the pandemic with multiresistant Gram-negative rods. Therefore, morbidity, mortality, and the associated economic burden are very likely to increase dramatically during the next decade [20]. Furthermore, with the current European financial crisis resulting in massive cuts in healthcare expenditure and medical research, we can expect multiresistant bacteria to spread more rapidly in hospitals.

### What are the causes of this frightening evolution?

The most important cause is that there has been a massive overuse of antibiotics worldwide across all ecosystems over the past decades, including humans, animals, aquaculture, and agriculture (Additional files 1 &2).

When selected silently by antibiotics, a hidden cross-transmission of resistant bacteria occurs daily, both in hospitals and communities. Compliance with hand hygiene practices is far from optimal in many healthcare settings, including hospitals and long-term care facilities [21], thus resulting in a continuous succession of small-size transmission events difficult to detect, as well as large outbreaks. Exchange of resistant bacteria via travel activities and patient transfers has led to a rapidly growing "resistance globalization" as recently exemplified by the spread of NDM-1 [8]. As a consequence, some countries recommend the preemptive isolation of patients admitted from outside their borders based on a suspicion of MDRO carriage in the same philosophy as the "Search and Destroy" programme in The Netherlands [22]. Cross-transmission occurs also in community settings (e.g., schools, families, daycare centres). Finally, hospital and community wastewater systems are an additional source for the dissemination of resistant bacteria.

In particular, the spread of antibiotic-resistant *Enterobacteriaceae *insensitive to third-generation cephalosporins and carbapenems poses a serious public health threat. Resistance to these beta-lactams is primarily attributed to the production of beta-lactamases, ESBLs and carbapenemases, respectively, and their coding genes located on mobile genetic elements (e.g., plasmids) facilitate intra- and interspecies transfer.

Many countries and healthcare facilities still lack effective antibiotic stewardship programmes [23]. Antibiotics continue to be considered as "ordinary" drugs and are prescribed freely by many different physicians, both in the community and in hospitals. In general, these physicians lack appropriate and rigorous training in infectious diseases and prescribe without any control or help. When national or local programmes do exist, they have often transient effects and require sustained and repeated incentives. As an example, the "Antibiotics are not automatic" ("Les antibiotiques, c'est pas automatique") programme launched in France in the early 2000s had a very positive effect during five years (23% overall decrease in consumption) [24], but consumption is now again on the rise [25]. Self-medication, an important driver of antibiotic overuse, is common, particularly in developing countries where antibiotics can be bought over the counter in pharmacies or in local market places, but it occurs also in Europe, mainly in southern and eastern countries [26]. Antibiotics are used in excess, particularly for common colds and upper respiratory tract syndromes that are mostly of viral origin. Direct sales via the internet are also increasing and difficult to control [27], including sales in some countries of illegal over-the-counter antibiotics and counterfeit drugs that may contain sub-optimal active antibiotic concentrations.

Simultaneously, the antibiotic pipeline is drying up for two reasons (Additional file 3): 1) it is intrinsically difficult to find new antibiotics with novel mechanisms of action; and 2) a high cost/benefit and risk/benefit ratio (length of development, low selling prices, and short treatments) discourage pharmaceutical companies from investment. Moreover, bacteria are rapidly developing when antibiotics are overused, which creates a dilemma for the profit-driven pharmaceutical industry. Therefore, new business models must be developed to encourage research and development arms of companies to engage in the discovery of new antibiotics, but these discussions have turned out to be very difficult. In addition, the financial crisis will probably increase the burden on tax payers and industry to invest in this field.

### Is there any national or international reaction to this threat?

Many national/international meetings, workshops, and task forces, as well as reports in the scientific literature and lay press, have been dedicated to this threat over the last decade, particularly in 2011, but often with a limited impact due to a lack of coordination [17,18,28-34]. Only a few developed countries worldwide [13-15,35-37] have managed to reduce antibiotic consumption in the community and/or successfully implemented hand hygiene campaigns in their hospitals, which have sometimes resulted, but not always, in a decrease in resistance. However, despite these efforts, resistance among Gram-negative rods has increased dramatically in parallel, while co-existing with good results for the decrease of MRSA infection [14].

Europe, in particular through the European Union (EU) Directorate General for Health and Consumers (DG SANCO; http://www.ec.europa.edu/dgs/health_consumer/) and the ECDC http://www.ecdc.europa.eu, supports and organizes comprehensive and well-validated surveillance networks for AMR and antibiotic consumption, which has allowed to monitor the impact of these interventions [6,38]. ECDC and the European Medicines Agency (EMA; http://www.ema.europa.eu) have jointly organized a meeting and urged pharmaceutical companies to accelerate the search for new antibiotics [17]. In 2009, a Transatlantic Taskforce for Antimicrobial Resistance (TATFAR; http://ecdc.europa.eu/en/activities/diseaseprogrammes/tatfar/pages/index.aspx?MasterPage=1) was established during the Swedish EU presidency to promote a mutual understanding of US and European activities and programmes related to AMR issues [39]. A list of 17 recommendations was generated in 2011, but with no incentives on how to reach its stated objectives and no mandate to address the global aspects of this problem http://ecdc.europa.eu/en/activities/diseaseprogrammes/tatfar/documents/210911_tatfar_report.pdf.

Regional and international networks or alliances have been developed also with various actions proposed, i.e., Action on Antibiotic Resistance (REACT; http://www.reactgroup.org), Alliance for the Prudent Use of Antibiotics (APUA; http://www.tufts.edu/med/apua/), the European Society for Clinical Microbiology and Infectious Diseases (ESCMID) Study Group on Antibiotic Policies (ESGAP; http://www.escmid.org/research_projects/study_groups/esgap/) [40], and the Alliance against MDRO [41]. Finally, in 2011, the World Health Organization (WHO) dedicated the World Health Day to the topic of antimicrobial resistance with the aim to highlight it as a global threat and to call for consolidated efforts to avoid regressing to the pre-antibiotic era http://http//:www.who.int/world-health-day 2011/en/index.html. Hopefully, this will be the starting point for tangible and sustained efforts by WHO through a worldwide campaign.

### Are we ready for a world without antibiotics?

The answer is clearly no! Today, antibiotics are critical to treat bacterial infections. Indeed, there are very few therapeutic compounds, if any, able to modulate the inflammatory burst during severe sepsis [42]. Anti-toxin therapy could represent a key component of the antibacterial armamentarium of the future, but it is too early to rely on this solution on a routine basis [43]. Antimicrobial peptides are deceiving, particularly when used intravenously [44]. Bacteriophages are tempting, but are not usable by the intravenous route and have not been carefully evaluated so far [45]. Resistance is also an issue with this strategy. Some plants or aromatic substances (e.g., essential oils) may have very interesting antibacterial and antitoxin activities, but again we are far from their use in daily practice [46]. Probiotics have been mentioned as a possible alternative, but could be considered today more as a complement than as a real therapeutic solution. Vaccination is certainly the most promising preventive strategy, but remains limited to a relatively small number of bacteria [47], although there are promising new vaccines entering phase III studies against *S. aureus *and *Clostridium difficile*. Without any doubt, antibiotics remain the cornerstone of antibacterial management and they are still acutely needed for the next generations. It is our duty to protect them.

### Can we rely on recent positive and transposable programmes?

The answer is clearly yes, but the examples are few. Education, legislation, and improved diagnosis can reduce antibiotic consumption. Several clinical trials at the community level, have shown that patient education can result in the decrease of the use of antibiotics [48]. The Patients for Patient Safety branch of the WHO Patient Safety Programme has shown that patients can and should have a very active role in making healthcare safer and will be examining how to integrate information on antibiotic resistance within its global training group for 2012 http://www.who.int/patientsafety/patients_for_patient/en/. The EU has established a strategy against AMR to encourage the prudent use of these agents in human medicine. Several countries have launched national campaigns to educate physicians and patients about antimicrobial misuse and the threat of resistance.

The French campaign, often considered as a model, exceeded expectations with a 23% reduction in the number of antibiotic prescriptions over the first five years [24]. However, nine years after the launch, there are still major concerns about the way in which physicians and patients in France prescribe and consume antibiotics. Despite the sharp reduction of antibiotic prescriptions observed, especially among children, France remains a high user of antibiotics, just behind Greece and Cyprus [6,7].

The Belgian Antibiotic Policy Coordination Committee (BAPCOC) organized several national campaigns, financially supported by the government. These multimedia campaigns, launched in 1999 and targeting the general public, resulted in a 36% decrease in antibiotic prescription in the community between 1999 and 2007 [35] and decreased antibiotic resistance in *Streptococcus pneumoniae *and *S. pyogenes*. The hand hygiene campaigns, launched in 2005 and targeting patients admitted to hospital and healthcare workers, resulted in an increase in hand hygiene compliance and alcohol-based handrub use in hospitals and decreased hospital-acquired (HA)-MRSA.

However, some national campaigns, e.g., Australia, England, Greece, and Spain, have failed to show a major impact on antibiotic prescriptions [35]. In the USA, some very positive results have been obtained for HA-MRSA, but not for CA-MRSA, VRE, and ESBL-carrying *Enterobacteriaceae *[48]. In Israel, some interesting results have been obtained in the use of antibiotics in children [22] and the successful containment of the pandemic with Gram-negative rods [49]. To control self-medication, the Chilean Ministry of Health has strictly enforced existing laws restricting the purchase of antibiotics without medical prescription since 1999. These regulatory measures have resulted in a 43% decrease in antimicrobial use in the outpatient setting, which represents a remarkable result [50]. Further interesting results from other countries were highlighted by poster presentations displayed during the meeting and are discussed by Jarlier et al in this issue [51].

Nevertheless, despite targeted information and awareness-raising campaigns, the general public has still preconceived ideas concerning antibiotics and their effects. For example, according to a Pan-European survey published in 2010, 53% of Europeans still believe that antibiotics kill viruses and 47% that they are effective against colds and influenza. Large variations between countries were observed and knowledge increased in countries with targeted media campaigns, such as Belgium. Education remains an immense challenge [52].

### Time for international coordinated actions to save antibiotics

In response to this global public health threat, 70 leading international experts formulated "The Pensières Antibiotic Resistance Call to Action" during a two-day meeting held in Annecy (France) in June 2011. Lectures were given on a wide range of topics with extensive and in-depth discussion. Each participant presented data and the results of country-specific intervention programmes targeted at the control of AMR and healthcare-associated infection, such as infection prevention and control and antibiotic stewardship strategies. Thirty-four posters provided for the first time a unique overview of actions and policies in place worldwide in 29 countries with an evaluation of their degree of efficacy. At the end of the meeting, participants were asked to rank a series of 25 actions linked to the topics highlighted using a multi-voting system [51].

A coordinated programme based on six main lines of action was defined as follows: 1) a worldwide upgrade in infection control practices to limit resistant bacteria cross-transmission; 2) a worldwide antibiotic stewardship strategy to decrease antibiotic pressure on bacteria; 3) the improved use of diagnostic techniques; 4) an acceleration in the discovery and development of new antibiotics, particularly targeting Gram-negative bacteria; 5) the acceleration of vaccine development programmes, and 6) a strong educational programme for both healthcare practitioners, consumers, and children.

The programme is conceived as a "bundle" whose different components should be implemented simultaneously. Implementing only one line of action or selecting only some components will very likely lead to failure. Such a multifaceted programme looks easy to implement, but is in fact a serious challenge. Although the multiple actors to involve have different backgrounds and interests, such as the hospital, community, and human and animal medicine, and may not work spontaneously together, cooperation between all is the key to success.

A strong political commitment at international, national, and local levels is of paramount importance to trigger such an ambitious programme. This is absolutely essential. Recent national or international programmes should be evaluated. Healthcare professionals will need the strong involvement of policy makers through to hospital managers to ensure its adoption. It will take many years to obtain significant results and we will certainly never return to the pre-antibiotic era where all pathogens were fully susceptible to antibiotics. But we have no choice and must preserve antibiotics for the next generations. We must realize also that such a programme is not aiming at simply saving money--and could even increase healthcare costs initially--but it will become eventually cost-effective when taking a long-range perspective.

### Effective infection control programmes must be implemented worldwide

The importance of a coordinated programme combining infection control with other actions in a rational and sustainable manner, e.g., antibiotic stewardship, must be strongly emphasized. Prevention of cross-transmission and epidemics must be based on a multifaceted strategy that should include appropriate screening policies, use of universal precautions, improved hand hygiene, particularly through the systematic recourse to alcohol-based handrub formulations (ABHRs), and specific contact precautions when appropriate (i.e., geographic isolation measures and cohorting). However, several of these measures remain controversial and costly. For instance, it remains unknown if specific isolation precautions are better than standard precautions if the latter are strictly and permanently applied, which so far is hard to obtain [53]. Rapid diagnostic methods are needed more than ever to detect patients colonized by MDROs and the Innovative Medicines Initiative (IMI; http://www.imi-europa.edu) is investing 15 million euros in the RAPP-ID project (Development of Rapid Point-of-Care Test Platforms for Infectious Diseases; http://www.rapp-id.eu) to develop new diagnostic tools for bloodstream infections, lower respiratory tract infections, and tuberculosis. It is to be hoped that some promising ongoing EU-funded projects will help to find new solutions, e.g., "Mastering hOSpital Antimicrobial Resistance" (MOSAR; http://www.Mosar-sic.org); "Impact of Specific Antibiotic Therapies on the prevalence of hUman host ResistaNt bacteria" (SATURN; http://www.saturn-project.edu); and "Resistance in Gram-Negative Organisms: Studying Intervention Strategies" (R-GNOSIS; http://www.r-gnosis.eu).

Successful measures for controlling MRSA are probably not sufficient to prevent the spread of ESBL or carbapenemases for several reasons: far higher bacterial load in the gut for Gram-negative rods; fecal excretion; dissemination through waste; transferable resistance genes on plasmid or transposons; lack of effective decolonization regimens; or the substantial role of antibiotic selection pressure by commonly misused drugs. Although MRSA bloodstream infections are decreasing in many European countries, infections due to ESBL-producing Gram-negative rods are increasing in these same countries. To succeed in combating these Gram-negative rods, there is a need to upgrade and tailor the prevention of cross-transmission outside hospitals (e.g., in nursing homes, families, daycare centres, and schools) and to take into account environmental aspects. Moreover, those actors with an important role to play, such as specialists in infection control and healthcare managers, have been somewhat paralyzed in front of the ESBL invasion, although sometimes simultaneously very active against MRSA or VRE.

The WHO Global Patient Safety Challenge "Clean Care is Safer Care" is a striking example of a programme that could provide guidance, boost hand hygiene promotion initiatives worldwide, including in developing countries. Proof of effectiveness of additional actions will hopefully be provided by ongoing studies [21]. Quality indicators are needed to assess the performance of hand hygiene procedures in hospitals, e.g., surrogate markers such as the volume of ABHR consumption (used in France, Belgium, and Germany) or, even better, the compliance rate with procedures (e.g., in Australia) as proposed by the WHO strategy [54]. Population migration and health tourism are unavoidable components of the modern era. Hospitals accepting international patients must follow excellent infection control practices and antibiotic stewardship policies in practice and not just on paper, including qualified and trained infection control teams and a hospital management willing to accept their recommendations.

### Active protection of antibiotics (part of the so-called "antibiotic stewardship")

Antibiotics are natural gifts belonging to humanity and strategies for their active protection must be developed in a philosophy of "sustainable development" [1]. A worldwide implementation of antibiotic stewardship programmes is of paramount importance [23,55]. This should be based on a multidisciplinary approach aimed at the optimal selection, dosage, and duration of antimicrobial treatment resulting in the best clinical outcome for treatment or prevention of infection with minimal toxicity to the patient, and minimal impact on subsequent resistance. The reason for the prescription and the planned duration of therapy (as well as diagnosis whenever possible) should be indicated on every patient chart. Indeed, in some countries, including the EU, many hospital physicians prescribe antibiotics without mentioning the reason in the patient notes [56]. Finally, some antibiotics should probably be reserved exclusively for human usage. However, there is no consensus with the veterinary world on this measure.

A concerted international programme should induce a marked decrease in the overall consumption of antibiotics in every sector of human and animal medicine, aquaculture, and agriculture. There is no specific culprit and all antibiotic prescribers must work together. A strong and sustained cooperation between healthcare professionals and consumers (antibiotic users) in an ecological and civic attitude is pivotal for the success of these programmes. Antibiotics must be considered as a specific class of drugs [40,57], a central concept that will have many consequences in terms of legislation, particularly at the European level. A major breakthrough would be obtained if antibiotics could be included in the United Nations Educational, Scientific and Cultural Organization (UNESCO) global heritage list for humanity to demonstrate and raise awareness of their long-term importance for human health http://www.whc.unesco.org. Finally, it is of paramount importance to realize that in many countries there is a very limited access to antibiotics, which impairs safety of care. A balance between appropriate usage and access to antibiotics is needed. These two actions are not mutually exclusive, but complementary.

### Diagnosis of bacterial infection and antibiotic resistance must be more rapid

Rapid diagnostic tests should be urgently developed to help physicians to target the organisms causing the infection. Physicians should not rely only on fever, which is very often due to non-bacterial infections, to prescribe antibiotics. Unfortunately, microbiology diagnostic techniques have not evolved much since Pasteur and others were able to grow bacteria at the end of the 19^th ^century, and many of their culture methods are still used today in our routine clinical diagnostic laboratories.

New rapid diagnostic tools, such as point-of-care testing or biomarkers, should be used more widely. These are already available for several microorganisms, including *C. difficile *and MRSA. Simple tests are available to detect *Streptococcus pyogenes *in the throat, but often not used by general practitioners (5 to 15% in adults; 30% in children [58]. Urinary sticks are sensitive enough to avoid treating most patients with a suspicion of urinary tract infection, particularly in long-term care facilities. Procalcitonin can help to differentiate viral and bacterial bronchitis [59].

The development of new tools should be encouraged to help clinicians not to treat patients with antibiotics when bacterial infection is ruled out or, conversely, to help them to prescribe the right antibiotic through rapid identification of the bacteria involved and its antibiotic susceptibility. Re-evaluation of therapy at days two or three should be systematic in all types of practice. Appropriate biomarkers [60] and therapeutic algorithms that include de-escalation strategies will help to reduce the length of therapy and optimize the choice of drugs [61]. After a long period where antibiotic therapy has been mostly empiric in many countries, including the USA, it is time to teach and treat infectious diseases based on diagnostic evidence. This will represent a dramatic change in our paradigm of care and a real challenge.

### New antibiotics are urgently needed and must be efficiently protected

WHO, TATFAR, the Infectious Diseases Society of America (IDSA), and European institutions, as well as healthcare professionals, have proposed measures and incentives to fix the broken antibiotic pipeline and encourage biotechnology and pharmaceutical companies to invest in the development of new antibacterial agents, particularly against Gram-negative bacteria. In 2010, IDSA launched a new initiative entitled "10 × 20" to mobilize key leaders, research institutions, and scientific associations to create an antibacterial research and development enterprise powerful enough to produce 10 new antibiotics by the year 2020 http://www.idsociety.org/10x20/[62]. Fast-track designation for the development of new drugs (similar to orphan drugs) to help get them earlier to the patient, high prices for antibiotics with a high value compared to others, including active protection and follow-up, are actions that will help to develop new drugs and protect them when marketed. Prolongation of antibiotic patents has been proposed, but remains controversial [63]. The IMI and the European Federation of Pharmaceutical Industries and Associations (EFPIA; http://www.efpia.org) are currently discussing mechanisms to jointly develop new antibiotics.

### A strong educational programme must be made available worldwide for both healthcare professionals and consumers

It is of paramount importance that both professionals and consumers understand that the two main causes of antibiotic resistance are their overuse in both humans, animals, and agriculture. A complicity between these two groups is key to the success of such a programme. We need also to provide information to children and to promote and establish large programmes such as the Pan-European e-Bug project http://www.e-bug-edu[64,65]. In turn, these children will teach their parents and other family members and will become more clever consumers of healthcare than we have ever been.

## Conclusion

We have overused and abused antibiotics in both humans and animals with huge variations between countries [66]. Today, we have regular and precise barometers to survey resistance levels and antibiotic consumption [67]. Resistance of bacteria to antibiotics has reached levels that place the human race in real danger. Immediate, vigorous, and coordinated measures must be taken worldwide to save and protect the erosion of existing antibiotics and facilitate the appearance of new and potent antibiotics, active in particular against Gram-negative bacilli [68,69]. This will need a profound change in the way we diagnose and treat infectious diseases [70]. Dramatic change will be needed also in the way we behave in hospitals and in the community concerning both antibiotic therapy and infection prevention and control measures [71]. Educational programmes targeting both healthcare professionals and consumers, including children, are urgently needed. A strong cooperation and complicity between healthcare providers, including researchers and consumers, is the real key to success.

## List of abbreviations

ABHRs: alcohol-based handrubs; AMR: antimicrobial resistance; APUA: Alliance for the Prudent Use of Antibiotics; BABCOC: Belgian Antibiotic Policy Coordination Committee; CA-MRSA: community-acquired methicillin-resistant *Staphylococcus aureus; *CDC: Centers for Disease Prevention and Control; DDD: defined daily dose; DG-SANCO: Directorate General for Health and Consumers; ECDC: European Centre for Disease Prevention and Control; EFPIA: European Federation of Pharmaceutical Industries and Associations; EMEA: European Medicines Agency; ESGAP: European Society for Clinical Microbiology and Infectious Diseases (ESCMID) Study Group on Antibiotic Policies; EU: European Union; ESBL: extended-spectrum beta-lactamase; FDA: Food and Drug Administration; HA-MRSA: hospital-acquired methicillin-resistant *Staphylococcus aureus*; HIV: human immunodeficiency virus; IDSA: Infectious Diseases Society of America; IMI: Innovative Medicines Initiative; MDROs: multidrug-resistant organisms; MOSAR: Mastering; hOSpital: Antimicrobial Resistance; MRSA: methicillin-resistant *Staphylococcus aureus; *NDM-1: New Delhi metallo-beta-lactamase 1; RAPP-ID: Development of Rapid Point-of-Care Test Platforms for Infectious Diseases; REACT: Action on Antibiotic Resistance; R-GNOSIS: Resistance in Gram-Negative Organisms: Studying Intervention Strategies; SATURN: Impact of Specific Antibiotic Therapies on the prevalence of hUman host ResistaNt bacteria; TATFAR: Transatlantic Taskforce for Antimicrobial Resistance; UK: United Kingdom; UNESCO: United Nations Educational, Scientific and Cultural Organization; USA: United States of America; VRE: vancomycin-resistant enterococci; WHO: World Health Organization

## Competing interests

Jean Carlet has served as a consultant for Biomerieux, Astra-Zeneca, Astellas, Thermo-Fisher, Da Voltera, and Aromatechnologies. John Conly has received honoraria from the Canadian Agency for Drugs and Technologies in Health for work as an expert reviewer and clinical expert, respectively, for projects on the role of rapid polymerase chain reaction (PCR) testing for MRSA in hospitalized patients, and the use of vancomycin or metronidazole for the treatment of *C. difficile *colitis. He has also received speaker's honoraria related to new antibacterial agents from Janssen-Ortho, Pfizer, and Astellas Pharma during the past five years. All other authors declare no competing interests.

## Authors' contributions

JC conceived and drafted the manuscript and produced the different versions, in particular the reference list. VJ and DP provided an extensive review of the first draft of the manuscript. DP, SH, HG, and AV reviewed the manuscript and provided important intellectual content. All attendees to the meeting were given the possibility to comment on the content. All authors have read and approved the final version of the manuscript.

### Disclaimer

The content of this paper expresses the views of the experts co-authoring it and in no way represents the position of their affiliations.

## Additional file 1

### Antibiotic use, misuse, and abuse

Half of all antibiotic consumption may be unnecessary and greatly contributes to increasing bacterial resistance [28]. **In Europe **(29 countries), the overall human consumption of antimicrobials was 3350 tons in 2007 [29]. Outpatient consumption varies widely from 11 defined daily doses (DDD) per 1000 inhabitants in The Netherlands to 34 DDD per 1000 inhabitants in Cyprus [38]. **In the USA**, 3300 tons of antibiotics were sold [18].

### Antibiotics are ineffective against viral infections

• But they are often prescribed for self-limiting illnesses, such as colds and influenza, caused by viruses that will not respond to antibacterial drugs.

• Diagnostic uncertainty is a key driver of drug misuse and overuse. Since classical laboratory methods, based on culture of the pathogenic agent, require 36-48 hours to provide results, few infections are accurately diagnosed.

• In the absence of a clear diagnosis, physicians often prescribe antibiotics just "to be on the safe side" or to prevent possible secondary bacterial infections.

• In addition, patients often put pressure on physicians. In a survey conducted in the USA, nearly half (48%) of respondents indicated that they expected an antibiotic when they visit a doctor [72] In another survey, more than 50% of French interviewees expected an antibiotic for the treatment of influenza-like illness [73].

### It is often falsely assumed that inappropriate use of antibiotics cannot harm

• According to the US Centers for Disease Control and Prevention (CDC), an estimated 150,000 cases per year present to US emergency departments for antimicrobial-related adverse events [74].

• Incorrect use of antibiotics accelerates AMR. In this respect, AMR is like pollution: it has so little immediately perceptible effect that in the absence of regulation, nothing changes [75].

**Patient compliance **with recommended treatment is another major problem

• Patients forget to take medication or may be unable to afford a full course. They tend to consider antibiotics as antipyretics that treat symptoms and stop taking them as soon as they feel better.

**Self-medication **is also an important driver of antimicrobial overuse

• It has been observed in the USA [76] and Europe [77,78], particularly for self-limiting illnesses mostly caused by viruses.

• It is especially prevalent in developing countries where antibiotics can be bought over the counter in pharmacies or even in the local market place.

• Sales via the internet drive self-medication; they are on the rise and difficult to control [27].

## Additional file 2

### Antibiotic use in animals: a major concern for public health and the environment

Resistance to antibiotics is increasing both in commensal and pathogenic bacteria, raising an emerging threat to public health and the environment. Antimicrobial administration to food animals is among the most important factors contributing to the selection of antimicrobial-resistant bacteria that can be transmitted from animals to humans. More than half of all antibiotics produced globally are used in animals [29]. In the USA alone, animal agriculture consumes 80% of all antibiotics used [79]. According to a first-ever estimate of the Food and Drug Administration (FDA), the amount of antibiotics sold for use in food animals in the USA was over 13,000 tons (29 million pounds) in 2009 [80]. The overall national sales of veterinary antimicrobials in 10 European countries was approximately 3500 tons of active substance in 2007 [29]. In 2009, French sales of veterinary antimicrobials were 1067 tons [81].

#### Antimicrobial use and animal husbandry

Antimicrobials are used by veterinary practitioners for the treatment and control of infectious diseases in a wide variety of farm and companion animal species. Antibiotic treatment of sick animals is common practice. When a certain percentage of farm animals or certain species (e.g., flocks of broiler chickens or salmon pens) are affected, the entire group is treated, including animals that are not infected. Sub-therapeutic levels of antibiotics are also administered to animals for the prevention of bacterial infections to compensate poor production practices, often without prescription.

Low levels of antibiotic agents are frequently added to animal feed for growth promotion in livestock (mostly in the production of pigs, broiler chickens, turkeys, and feedlot cattle) [82]. This is particularly problematic because antibiotic growth promoters are used without veterinary prescriptions or administered for long periods of time at sub-therapeutic concentrations to entire groups or herds of animals. This favours the selection and spread of resistant bacteria [83].

#### National legislation

On January 1 2006, the EU banned the feeding of all antibiotics and related drugs to livestock for growth promotion purposes [84]. The USA has not yet implemented similar control policies for antibiotic use in animal agriculture. However, a recently issued FDA Guidance to Industry called for the use of antibiotics in food-producing animals only when needed to ensure animal health, including phasing in veterinary oversight and consultation, and has attracted growing support within Congress for new legislation [85,86].

### Transmission of resistant bacteria from animals to humans

Widespread use of antimicrobials for disease control and growth promotion in animals has been paralleled by an increase in resistance in those bacteria in animals. Resistant bacteria then spread among groups of animals, including fish, or to the local environment (adjacent soil, air, and water) through the spreading of manure.

-- *Through long-term survival and transfer of resistant genes to the resident flora *[87] Studies carried out in The Netherlands have shown that the proportion of resistant bacteria containing antibiotic resistance genes in the soil has significantly increased since 1940 [88].

-- *Through direct contact between farm animals and humans (e.g., farmers, farm visitors) *The same strains of MRSA have been found in livestock and livestock workers in The Netherlands, Italy, Canada and the USA [89-91].

-- *Through contaminated food*

Although correct cooking kills bacteria, contamination can occur through improper handling before cooking. Many of the antimicrobial-resistant *E. coli *strains that cause urinary tract and bloodstream infections in humans appear likely to have been acquired from contaminated retail meat.

In The Netherlands, 94% of a representative sample of chicken retail meat was contaminated with ESBL-producing *E. coli *isolates, of which 39% were also found in human clinical samples tested in 31 microbiological laboratories [92,93]. An association between the approval of fluoroquinolones for use in food-producing animals and the development of fluoroquinolone-resistant *Salmonella *and *Campylobacter *in animals and humans has been observed in several countries [31,94-96]. Reports of the spread of multidrug resistant *Salmonella Schwarzengrund *from chickens to humans in Thailand and from imported Thai food products to humans in Denmark and the USA [97].

### Use of antibiotics in food animals may result in the deposition of residues in animal products and the environment

• Consumption of antibiotic residues represents a potential threat to human health, through direct toxicity, allergic reactions, or alteration of the bacterial flora present in the human digestive tract [98].

• To safeguard humans from exposure to antibiotic-added food, a withholding period must be observed until the residues are no longer detected before the animal or animal products can be processed. Heavy responsibility is placed on the veterinarian and livestock producer to observe the period of withdrawal. In Europe, rapid tests are regularly performed to check the absence of antibiotic residues in food.

• Eliminating the unnecessary usage of antibiotics implies a change in mindset, integrating both long-term public health concerns and productivity. This involves everyone -- from governments to producers to the consumer. To stem the rising threat of resistant bacteria to human health, there is an urgent need for regulation of antibiotic usage in animals at the global level.

## Additional file 3

### The antibiotic pipeline is running dry

In the past, the discovery of potent new classes of antimicrobials allowed to provide therapeutic options for newly emerging AMR. During the 30 years following the introduction of penicillin, scientists discovered a wide range of antimicrobials to treat bacterial diseases. By the early 1970s, 11 distinct antibiotic classes and more than 270 antibiotics had been brought into clinical use [99].

The process of novel antimicrobial discovery has slowed to a virtual standstill. Most antimicrobials introduced since the early 1970s have been chemical modifications of previously discovered classes of drugs [40]. The promise of genomics in discovering new antibiotic entities has remained largely unfulfilled to date.

#### Pharmaceutical companies have curtailed their anti-infective research programmes

• Of the 15 companies with previous had antibiotic discovery programmes, only 5 still maintain an active research and development capacity in antibiotics [32].

• According to two recent reports from IDSA [33] and the ECDC and EMEA [17], there are only a few candidates in company pipelines.

• Only 15 antibiotics under development (mostly in the early phases) present a new mechanism of action with the potential to meet the challenge of multidrug resistance. Of these, only two, both in the early development phase, may be active against multidrug- resistant Gram-negative bacteria, a group of bacteria causing serious therapeutic concerns due to their increasingly high resistance to antibiotics.

### Why is the antibiotic pipeline drying up?

The discovery and development of new antimicrobials is an expensive and time-consuming process. Pharmaceutical companies must prioritize competing projects and antibiotic development has a lower priority than other competing drugs in the portfolio.

• In the late 1960s, infectious diseases were thought to be conquered, opening the way for a shift in resources to chronic conditions, such as cancer and cardiovascular diseases.

• The limited duration of antibiotic treatments makes them less profitable than other drugs prescribed for years to treat chronic conditions, such as hypertension and diabetes.

• There is strong competition with other drugs already on the market. While resistance is an emerging problem, low-priced generic antibiotics on the market are still effective in treating most infections and are used as first-line therapy.

• New antibiotics may be kept as last-resort treatments, resulting in low sales for companies.

• New antimicrobials can also have a limited lifespan because of the development of resistance.

• Modifications in regulatory procedures have been perceived as having created an "unfriendly" environment. Regulators have been demanding demonstrations of the relative efficacy of new antibiotics *versus *those already registered within tighter statistical parameters, i.e., shifting from "non-inferiority" to "superiority" trials [40,100].

## Supplementary Material

Additional file 1 **Antibiotic use, misuse, and abuse**. Supplementary list of the main issues discussed (references [72-78]). Click here for file

Additional file 2 **Antibiotic use in animals: a major concern for public health and the environment**. Supplementary list of the main issues discussed (references [79-98]). Click here for file

Additional file 3 **The antibiotic pipeline is running dry**. Supplementary list of the main issues discussed (references [99,100]). Click here for file
