# Supplementary material for: Ready for a world without antibiotics? The Pensières Antibiotic Resistance Call to Action
Source: Antimicrob Resist Infect Control. 2012 Feb 14;1:11. doi: 10.1186/2047-2994-1-11 (PMC3436635; doi:10.1186/2047-2994-1-11)
Supplement: Additional file 1 — Antibiotic use, misuse, and abuse. Supplementary list of the main issues discussed (references [72-78]). [file 2047-2994-1-11-S1.DOC]

**Panel 1: Antibiotic use, misuse, and abuse**

Half of all antibiotic consumption may be unnecessary and greatly contributes to increasing bacterial resistance [28]. **In Europe** (29 countries), the overall human consumption of antimicrobials was 3350 tons in 2007 [29]. Outpatient consumption varies widely from 11 defined daily doses (DDD) per 1000 inhabitants in The Netherlands to 34 DDD per 1000 inhabitants in Cyprus [38]. **In the USA**, 3300 tons of antibiotics were sold (18).

**Antibiotics are ineffective against viral infections**

- But they are often prescribed for self-limiting illnesses, such as colds and influenza, caused by viruses that will not respond to antibacterial drugs.
  - Diagnostic uncertainty is a key driver of drug misuse and overuse. Since classical laboratory methods, based on culture of the pathogenic agent, require 36-48 hours to provide results, few infections are accurately diagnosed.
- In the absence of a clear diagnosis, physicians often prescribe antibiotics just “to be on the safe side” or to prevent possible secondary bacterial infections.
- In addition, patients often put pressure on physicians. In a survey conducted in the USA, nearly half (48%) of respondents indicated that they expected an antibiotic when they visit a doctor [72] In another survey, more than 50% of French interviewees expected an antibiotic for the treatment of influenza-like illness [73].

**It is often falsely assumed that inappropriate use of antibiotics cannot harm**

- According to the US Centers for Disease Control and Prevention (CDC), an estimated 150,000 cases per year present to US emergency departments for antimicrobial-related adverse events [74].
- Incorrect use of antibiotics accelerates AMR. In this respect, AMR is like pollution: it has so little immediately perceptible effect that in the absence of regulation, nothing changes [75].

**Patient compliance** with recommended treatment is another major problem

- Patients forget to take medication or may be unable to afford a full course. They tend to consider antibiotics as antipyretics that treat symptoms and stop taking them as soon as they feel better.

**Self-medication** is also an important driver of antimicrobial overuse

- It has been observed in the USA [76] and Europe [77,78], particularly for self-limiting illnesses mostly caused by viruses.
- It is especially prevalent in developing countries where antibiotics can be bought over the counter in pharmacies or even in the local market place.
- Sales via the internet drive self-medication; they are on the rise and difficult to control [27].
