# Supplementary material for: Ready for a world without antibiotics? The Pensières Antibiotic Resistance Call to Action
Source: Antimicrob Resist Infect Control. 2012 Feb 14;1:11. doi: 10.1186/2047-2994-1-11 (PMC3436635; doi:10.1186/2047-2994-1-11)
Supplement: Additional file 2 — Antibiotic use in animals: a major concern for public health and the environment. Supplementary list of the main issues discussed (references [79-98]). [file 2047-2994-1-11-S2.DOC]

| **Panel 2: Antibiotic use in animals: a major concern for public health and the environment**  Resistance to antibiotics is increasing both in commensal and pathogenic bacteria, raising an emerging threat to public health and the environment. Antimicrobial administration to food animals is among the most important factors contributing to the selection of antimicrobial-resistant bacteria that can be transmitted from animals to humans. More than half of all antibiotics produced globally are used in animals [29]. In the USA alone, animal agriculture consumes 80% of all antibiotics used [79]. According to a first-ever estimate of the Food and Drug Administration (FDA), the amount of antibiotics sold for use in food animals in the USA was over 13,000 tons (29 million pounds) in 2009 [80]. The overall national sales of veterinary antimicrobials in 10 European countries was approximately 3500 tons of active substance in 2007 [29]. In 2009, French sales of veterinary antimicrobials were 1067 tons [81].  *Antimicrobial use and animal husbandry*  Antimicrobials are used by veterinary practitioners for the treatment and control of infectious diseases in a wide variety of farm and companion animal species. Antibiotic treatment of sick animals is common practice. When a certain percentage of farm animals or certain species (e.g., flocks of broiler chickens or salmon pens) are affected, the entire group is treated, including animals that are not infected. Sub-therapeutic levels of antibiotics are also administered to animals for the prevention of bacterial infections to compensate poor production practices, often without prescription.  Low levels of antibiotic agents are frequently added to animal feed for growth promotion in livestock (mostly in the production of pigs, broiler chickens, turkeys, and feedlot cattle) [82]. This is particularly problematic because antibiotic growth promoters are used without veterinary prescriptions or administered for long periods of time at sub-therapeutic concentrations to entire groups or herds of animals. This favours the selection and spread of resistant bacteria [83].  *National legislation*  On January 1 2006, the EU banned the feeding of all antibiotics and related drugs to livestock for growth promotion purposes [84]. The USA has not yet implemented similar control policies for antibiotic use in animal agriculture. However, a recently issued FDA Guidance to Industry called for the use of antibiotics in food-producing animals only when needed to ensure animal health, including phasing in veterinary oversight and consultation, and has attracted growing support within Congress for new legislation [85,86]. |
| --- |

**Panel 2 (contd.)**

**Transmission of resistant bacteria from animals to humans**

Widespread use of antimicrobials for disease control and growth promotion in animals has been paralleled by an increase in resistance in those bacteria in animals. Resistant bacteria then spread among groups of animals, including fish, or to the local environment (adjacent soil, air, and water) through the spreading of manure.

— *Through long-term survival and transfer of resistant genes to the resident flora* [87]

Studies carried out in The Netherlands have shown that the proportion of resistant

bacteria containing antibiotic resistance genes in the soil has significantly increased

since 1940 [88].

— *Through direct contact between farm animals and humans (e.g., farmers, farm visitors)*

The same strains of MRSA have been found in livestock and livestock workers in The Netherlands, Italy, Canada and the USA [89-91].

— *Through contaminated food*

#### Although correct cooking kills bacteria, contamination can occur through improper handling before cooking. Many of the antimicrobial-resistant *E. coli* strains that cause urinary tract and bloodstream infections in humans appear likely to have been acquired from contaminated retail meat.

In The Netherlands, 94% of a representative sample of chicken retail meat was contaminated with ESBL-producing *E. coli* isolates, of which 39% were also found in human clinical samples tested in 31 microbiological laboratories [92,93]. An association between the approval of fluoroquinolones for use in food-producing animals and the development of fluoroquinolone-resistant *Salmonella* and *Campylobacter* in animals and humans has been observed in several countries [31,94-96]. Reports of the spread of multidrug resistant *Salmonella Schwarzengrund* from chickens to humans in Thailand and from imported Thai food products to humans in Denmark and the USA [97].

Use of antibiotics in food animals may result in the deposition of residues in animal products and the environment

- Consumption of antibiotic residues represents a potential threat to human health, through direct toxicity, allergic reactions, or alteration of the bacterial flora present in the human digestive tract [98].
- To safeguard humans from exposure to antibiotic-added food, a withholding period must be observed until the residues are no longer detected before the animal or animal products can be processed. Heavy responsibility is placed on the veterinarian and livestock producer to observe the period of withdrawal. In Europe, rapid tests are regularly performed to check the absence of antibiotic residues in food.
- Eliminating the unnecessary usage of antibiotics implies a change in mindset, integrating both long-term public health concerns and productivity. This involves everyone — from governments to producers to the consumer. To stem the rising threat of resistant bacteria to human health, there is an urgent need for regulation of antibiotic usage in animals at the global level.
