# Supplementary material for: Ready for a world without antibiotics? The Pensières Antibiotic Resistance Call to Action
Source: Antimicrob Resist Infect Control. 2012 Feb 14;1:11. doi: 10.1186/2047-2994-1-11 (PMC3436635; doi:10.1186/2047-2994-1-11)
Supplement: Additional file 3 — The antibiotic pipeline is running dry. Supplementary list of the main issues discussed (references [99,100]). [file 2047-2994-1-11-S3.DOC]

**Panel 3: The antibiotic pipeline is running dry**

In the past, the discovery of potent new classes of antimicrobials allowed to provide therapeutic options for newly emerging AMR. During the 30 years following the introduction of penicillin, scientists discovered a wide range of antimicrobials to treat bacterial diseases. By the early 1970s, 11 distinct antibiotic classes and more than 270 antibiotics had been brought into clinical use [99].

The process of novel antimicrobial discovery has slowed to a virtual standstill. Most antimicrobials introduced since the early 1970s have been chemical modifications of previously discovered classes of drugs [40]. The promise of genomics in discovering new antibiotic entities has remained largely unfulfilled to date.

*Pharmaceutical companies have curtailed their anti-infective research programmes*

• Of the 15 companies with previous had antibiotic discovery programmes, only 5 still maintain an active research and development capacity in antibiotics [32].

• According to two recent reports from IDSA [33] and the ECDC and EMEA [17], there are only a few candidates in company pipelines.

• Only 15 antibiotics under development (mostly in the early phases) present a new

mechanism of action with the potential to meet the challenge of multidrug resistance. Of these, only two, both in the early development phase, may be active against multidrug- resistant Gram-negative bacteria, a group of bacteria causing serious therapeutic concerns due to their increasingly high resistance to antibiotics.

**Why is the antibiotic pipeline drying up?**

The discovery and development of new antim**i**crobials is an expensive and time-consuming process. Pharmaceutical companies must prioritize competing projects and antibiotic development has a lower priority than other competing drugs in the portfolio.

- In the late 1960s, infectious diseases were thought to be conquered, opening the way for a shift in resources to chronic conditions, such as cancer and cardiovascular diseases.
- The limited duration of antibiotic treatments makes them less profitable than other drugs prescribed for years to treat chronic conditions, such as hypertension and diabetes.
- There is strong competition with other drugs already on the market. While resistance is an emerging problem, low-priced generic antibiotics on the market are still effective in
   treating most infections and are used as first-line therapy.
- New antibiotics may be kept as last-resort treatments, resulting in low sales for companies.
- New antimicrobials can also have a limited lifespan because of the development of
   resistance.
- Modifications in regulatory procedures have been perceived as having created an
  “unfriendly” environment. Regulators have been demanding demonstrations of the relative efficacy of new antibiotics *versus* those already registered within tighter statistical parameters, i.e., shifting from “non-inferiority” to “superiority” trials [40,100].
